# Variation in Macrophage Phagocytosis of *Streptococcus agalactiae* Does Not Reflect Bacterial Capsular Serotype, Multilocus Sequence Type, or Association with Invasive Infection

**DOI:** 10.20411/pai.v3i1.233

**Published:** 2018-05-18

**Authors:** Lisa M. Rogers, Jennifer A. Gaddy, Shannon D. Manning, David M. Aronoff

**Affiliations:** 1 Division of Infectious Diseases, Department of Medicine, Vanderbilt University Medical Center, Nashville, Tennessee; 2 Department of Pathology, Microbiology, and Immunology, Vanderbilt University Medical Center, Nashville, Tennessee; 3 Department of Microbiology and Molecular Genetics, Michigan State University, East Lansing, Michigan; 4 Department of Obstetrics and Gynecology, Vanderbilt University Medical Center, Nashville, Tennessee

**Keywords:** Neonatal sepsis, innate immunity, macrophages, Gram-positive bacteria, diabetes

## Abstract

**Background:**

Group B *Streptococcus* (GBS) is an encapsulated Gram-positive coccus that is an important cause of infections in adults with chronic medical conditions, pregnant women, and neonates. GBS causes a range of clinical syndromes, from asymptomatic colonization to deep-seated invasive and highly lethal infections. Macrophages are important sentinels of innate immunity, protecting host tissues from infection when bacteria advance beyond cutaneous or mucosal barriers. We hypothesized that the capacity for macrophages to phagocytose unopsonized GBS would vary across distinct clinical strains, and such differences would reflect serotype diversity.

**Methods:**

A high-throughput screen using the phorbol ester-differentiated THP-1 macrophage-like human cell line was used to quantify phagocytosis of a diverse group of 35 different human clinical isolates of GBS representing a wide variety of capsular serotypes. Validation studies were conducted using human primary phagocytes.

**Results:**

Phagocytosis of GBS differed widely across clinical isolates but this was not related to capsular serotype, genetic sequence type, pilus type, or clinical source of the GBS isolate (colonizing or invasive strain).

**Conclusions:**

Structural and/or biochemical differences among diverse GBS strains are reflected in a diverse capacity for macrophages to ingest them through non-opsonic phagocytosis. Mechanisms explaining these differences are not clear.

**STANDFIRST**

Variation in macrophage phagocytosis across diverse GBS isolates

Group B *Streptococcus* (GBS) is a β-hemolytic, encapsulated, Gram-positive bacterium with a propensity to cause infections in adults with chronic medical conditions, pregnant women, and neonates [1, 2]. GBS causes a range of illnesses, from asymptomatic colonization to invasive disease. The basis of this clinical variability is unclear, though differences across GBS strains clearly matter, as certain capsular serotypes and genetic subtypes are more common causes of invasive disease than others [3]. Of the 10 known capsular serotypes, five cause the vast majority of invasive infections [3]. A variety of bacterial virulence factors influences the risk for, and severity of, infections [4]. The presence (and/or expression) of such factors can vary widely across distinct GBS strains, and this likely explains many differences in colonization, immune evasion, and tissue invasion [4]. On the other hand, host determinants of vulnerability to infection remain incompletely defined, though the presence of anti-capsular antibodies clearly influences risk and provides the rationale for developing anti-capsular vaccines [5]. Anti-capsular immunoglobulins generated by previously exposed (or immunized) hosts can augment the function of innate phagocytes such as tissue macrophages. However, in non-immune persons with low levels of preexisting antibodies, macrophages (and other phagocytes) must internalize bacteria that are either opsonized by non-specific complement proteins or unopsonized bacteria. Because the sialylated polysaccharide capsule of GBS prevents host complement deposition on the bacterial surface [6], the capacity for macrophages to internalize unopsonized GBS bacteria is likely important. It is possible that diversity in the ability of GBS strains to cause disease reflects differences in macrophage evasion. We hypothesized that the capacity for macrophages to phagocytose unopsonized GBS would vary across distinct clinical strains, and such differences would reflect serotype diversity, possibly helping to explain why GBS demonstrates such distinct serotype diversity in its propensity to cause infection.

To test this hypothesis we utilized a high-throughput assay of bacterial phagocytosis using the human monocyte-like THP-1 cell line (ATCC TIB-202), differentiated into a macrophage-like cells with phorbol 12-myristate 13-acetate (PMA) [7, 8] as we have described for *S. pyogenes* [9, 10]. A diverse collection of 35 clinical GBS isolates (Table 1) was heat-inactivated, washed, resuspended, and labeled with the fluorescent dye fluorescein isothiocyanate (FITC) for use in the phagocytosis assay. In addition to facilitating assay standardization, bacterial heat inactivation limited the likelihood that newly synthesized or secreted mediators would influence macrophage behavior. All invasive GBS strains were originally recovered from neonatal blood or cerebral spinal fluid [11], while the colonizing strains were recovered via vaginal/rectal swabs from women during and after pregnancy [12]. Bacteria were incubated with PMA-differentiated THP-1 cells for three hours (at a ratio of 150 bacteria per cell) prior to measuring intracellular fluorescence as described [9, 10, 13]. Growth curves for each bacterial isolate obtained in tandem with heat-inactivation and fluorescent labeling allowed the generation of standard curves to estimate the number of fluorescently labeled bacteria internalized on a per-macrophage basis (Figure 1).

**Table 1:** Molecular characteristics and source of strains evaluated in this study.

Strain ID	Strain Type	Sequence Type (ST)	Pilus Island Profile (PI)	Molecular Serotype	Clinical Presentation
GB00112	Colonizing	ST-17	PI-2b	cpsIII	Vaginal/rectal colonization
GB00557	Colonizing	ST-17	PI-2b	cpsIII	Vaginal/rectal colonization
GB00663	Colonizing	ST-19	PI-2a	cpsIII	Vaginal/rectal colonization
GB00590	Colonizing	ST-19	Unknown	cpsIII	Vaginal/rectal colonization
GB00012	Colonizing	ST-1	Unknown	cpsV	Vaginal/rectal colonization
GB00571	Colonizing	ST-19	PI-2a	cpsIII	Vaginal/rectal colonization
GB00653	Colonizing	ST-12	Unknown	cpsII	Vaginal/rectal colonization
GB00020	Colonizing	ST-1	Unknown	cpsV	Vaginal/rectal colonization
GB00084	Colonizing	ST-1	PI-2b	cpsVIII	Vaginal/rectal colonization
GB00555	Colonizing	ST-12	Unknown	cpsIb	Vaginal/rectal colonization
GB00279	Colonizing	ST-23	Unknown	cpsII	Vaginal/rectal colonization
GB00097	Colonizing	ST-17	PI-2b	cpsIII	Vaginal/rectal colonization
GB00291	Colonizing	ST-12	Unknown	cpsII	Vaginal/rectal colonization
GB00285	Colonizing	ST-12	Unknown	cpsIb	Vaginal/rectal colonization
GB00651	Colonizing	ST-19	PI-2a	cpsIb	Vaginal/rectal colonization
GB00561	Colonizing	ST-19	PI-2a	cpsV	Vaginal/rectal colonization
NEM316	Invasive	ST-23	PI-2a	cpsIII	EOD/sepsis
GB00079	Invasive	ST-19	PI-2a	cpsIII	EOD/sepsis
GB00418	Invasive	ST-17	PI-2b	cpsIII	EOD/sepsis
GB01455	Invasive	ST-12	Unknown	cpsII	Stillbirth
GB00411	Invasive	ST-17	PI-2b	cpsIII	EOD/sepsis
GB00377	Invasive	ST-19	PI-2a	cpsIII	EOD/sepsis
GB00037	Invasive	ST-1	Unknown	cpsV	EOD/sepsis
GB00033	Invasive	ST-23	PI-2a	cpsIa	EOD/sepsis
GB00036	Invasive	ST-19	PI-2a	cpsIII	EOD/sepsis
GB00310	Invasive	ST-1	PI-2a	cpsV	EOD/sepsis
GB00686	Invasive	ST-1	Unknown	cpsV	Stillbirth
GB00910	Invasive	ST-12	Unknown	cpsII	EOD/sepsis
GB01007	Invasive	ST-19	PI-2a	cpsIII	Stillbirth
GB00390	Invasive	ST-23	Unknown	cpsIa	EOD/sepsis/meningitis
GB00374	Invasive	ST-12	Unknown	cpsIb	EOD/sepsis
GB00438	Invasive	ST-12	Unknown	cpsIb	LOD/sepsis
GB01454	Invasive	ST-1	Unknown	cpsV	Stillbirth
GB00045	Invasive	ST-17	PI-2b	cpsIII	EOD/sepsis
GB00066	Invasive	ST-19	PI-2a	cpsIII	EOD/sepsis

EOD = early onset disease; LOD = late onset disease

**Table 1: Clinical characteristics of group B streptococcus (*S. agalactiae*) isolates.** Strain ID, clinical type, sequence type (ST), pilus island (PI) profile, molecular serotype, and clinical presentation data on 35 clinical isolates screened in our phagocytosis assay.

**Figure 1.**
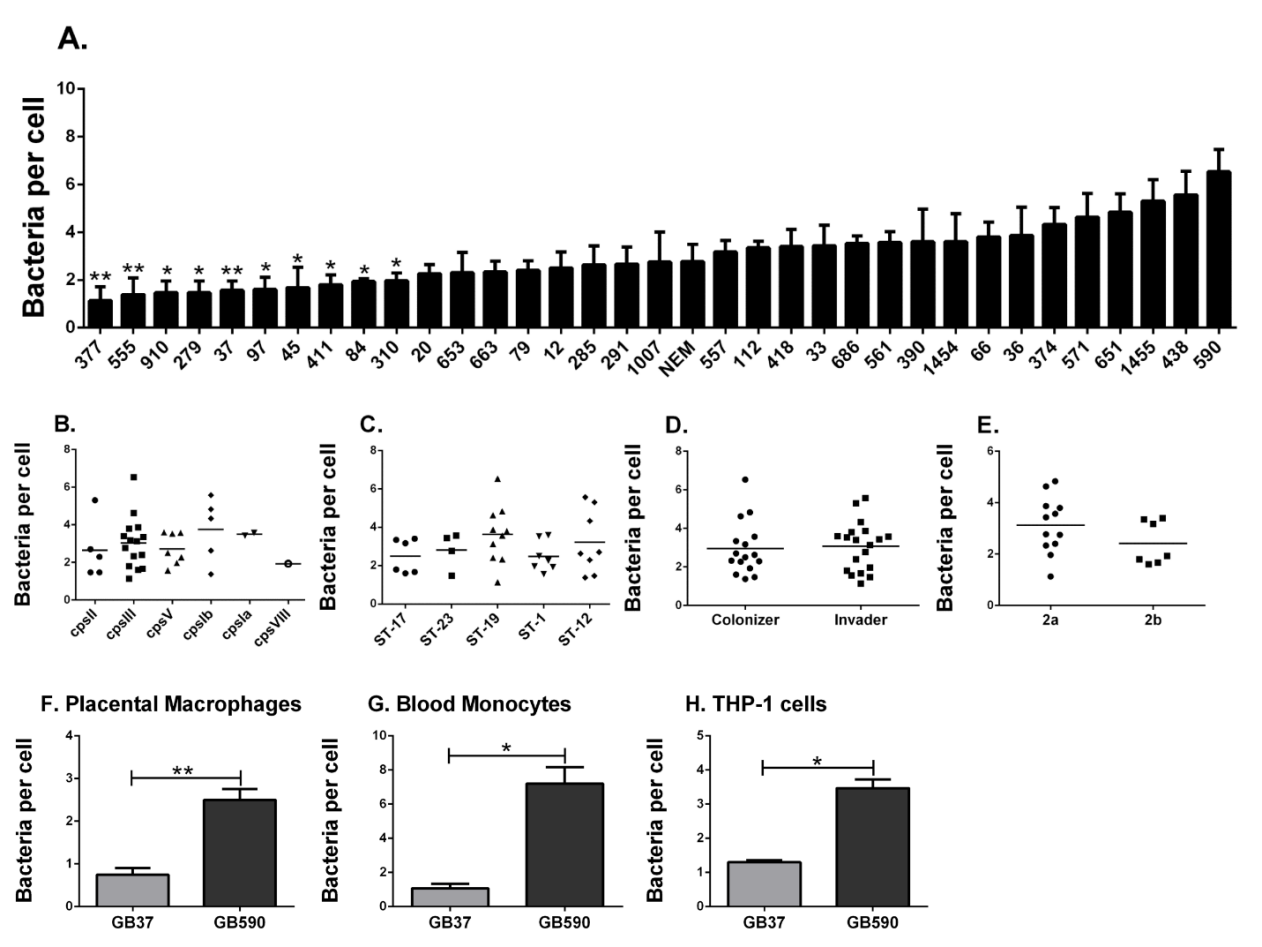
**Phagocytosis of clinical group B streptococcal isolates by human macrophages**. (A) THP-1 cells were assessed in their ability to phagocytose 35 diverse clinical strains of GBS. Cells were challenged with heat killed, FITC-labeled GBS at a MOI of 150:1. Phagocytosis of GBS was quantified by fluorometry after 3 hr. Data are represented as mean ± standard error of the mean (SEM); n = 3-5 for each. *P < 0.05 and **P < 0.01 by 1-way ANOVA and Tukey's post-test compared with phagocytosis of the GB590 strain. GBS is stratified by (B) molecular serotype (capsule type), (C) sequence type, (D) clinical presentation, or (E) pilus type. Phagocytosis of GB37 and GB590 was confirmed in (F) human primary placental macrophages, (G) human peripheral blood monocytes, and (H) new independent experiments using THP-1 cells and just these two strains. Data are represented as mean ± SEM of at least three independent experiments. *P < 0.05 by paired student t-test (two-tailed).

As illustrated (Figure 1A), we measured a wide range of bacterial phagocytosis by the number of THP-1 cells, ranging from 1.133 ± 0.584 bacteria/cell (± standard error of the mean) to 6.533 ± 0.935 bacteria per cell. These differences were significant by ANOVA, with significance observed specifically between the most-highly internalized strain (GB590) compared to 10 of the least-phagocytosed isolates (Figure 1A). These data support the first part of our hypothesis. Interestingly, however, when stratified by capsular serotype (Figure 1B), multilocus sequence type (Figure 1C as described in [14]), or strain source (Figure 1D), no significant differences were observed. For the subset of strains of GBS for which we had information related to the presence or absence of the gene encoding the pilus backbone protein Spb1 (SAN1518, PI-2b) [15], this feature did not associate with differences in macrophage uptake (Figure 1E). In summary, the data fail to support the second part of our hypothesis and suggest that variables other than capsular serotype, genetic sequence type, pilus type, or clinical source of the GBS strains explain the diverse capacity for THP-1 cells to internalize the bacteria. Future studies might evaluate whether these bacterial characteristics have more of an influence on macrophage phagocytosis mediated by opsonins, such as complement or immunoglobulin.

Because large screening studies are susceptible to false discovery (type I error), we sought to validate findings from the broad screen (Figure 1A) by comparing a single highly phagocytosed GBS strain to a single poorly internalized GBS strain head-to-head. The same fluorescence assay was utilized, but we conducted experiments in either PMA-differentiated THP-1 cells or two types of primary human phagocytes (Figures 1F-H). We used GBS strain GB590 because it was the most highly internalized bacterium in our screen, and strain GB37, a poorly internalized isolate. GB37 is notable because we have previously characterized it as a highly virulent strain *in vivo* and have sequenced the genome [16, 17]. Interestingly, GB37 was also found to be more virulent in systemic infection models in mice than GB590 [18], although the relation of *in vivo* behavior to our current *in vitro* findings is not clear. As shown in Figure 1F, the difference in phagocytosis observed in our initial broad screen was confirmed in human, primary placental macrophages, obtained from term, non-laboring placentas obtained at the time of Cesarean section, as previously described [10, 19]. This significant difference in phagocytosis was also observed in human primary blood monocytes (isolated as we have described [20]; Figure 1G) and repeated again in PMA-differentiated THP-1 cells (Figure 1H).

This study demonstrates that human macrophage-like cells (PMA-differentiated THP-1 cells) and primary human phagocytes phagocytose diverse GBS strains differentially. The basis of this does not appear to reflect capsular serotype, genomic sequence type, or the association of the GBS isolates with invasive disease. Future studies are needed to define the bacterial determinants of this variability, including the possibility that differences in sialic acid modifications of the GBS capsule might explain the varied macrophage responses, through interactions with host cell siglec receptors (reviewed in [21]). Interestingly, a previous study of 163 GBS isolates from different phylo-genetic backgrounds assessed non-opsonic phagocytosis assays using the mouse J774A.1 macrophage-like cell line [22]. As in our present study, they noticed a wide range of phagocytic activity across strains. However, their results demonstrated a relationship between phagocytosis and the presence of the gene encoding the pilus backbone protein, Spb1, with Spb1 expression by GBS being associated with greater phagocytosis [22]. Our data (Figure 1E) do not concur with that study. The reasons why we did not observe a similar impact of Sbp1 on phagocytosis are unclear, but might reflect technical differences between the studies. The prior study used live bacteria, wherein phagocytosis *per se* can be confounded by intracellular killing by the host phagocyte. Thus, differences in the recovery of live cells from macrophages may represent a composite endpoint related to both internalization of bacteria and intracellular killing. On the other hand, we used nonviable (heat-inactivated) bacteria, which removes the influence of bacterial killing as a confounder but also eliminates active bacterial processes that might influence host cell engagement and internalization. Our studies also used different macrophage cell lines. The previous study used a mouse tumor cell line [23], while we used a differentiated human monocytic leukemia cell line [8]. Future studies are needed to clarify the basis of the differences between these two studies.

In summary, this study demonstrates that structural and/or biochemical differences among diverse GBS strains are reflected in a diverse capacity for macrophages to ingest them through non-opsonic phagocytosis. The exact mechanisms explaining these differences are not clear. The extent to which the findings from this study are relevant to the pathogenesis of human infection is also unclear.

## ETHICS STATEMENT

This work involved using deidentified human cells and tissues for which specific consent was not required, as determined by the Vanderbilt University Institutional Review Board, which approved the studies with human blood and placenta. Placental samples were provided by the Cooperative Human Tissue Network at Vanderbilt University, which is funded by the National Cancer Institute.
